# Structural Characterization of Nanocellulose/Fe_3_O_4_ Hybrid Nanomaterials

**DOI:** 10.3390/polym14091819

**Published:** 2022-04-29

**Authors:** Aleksandra Janićijević, Vera P. Pavlović, Danijela Kovačević, Marko Perić, Branislav Vlahović, Vladimir B. Pavlović, Suzana Filipović

**Affiliations:** 1 Academy of Applied Technical Studies Belgrade, Katarine Ambrozic 3, 11000 Belgrade, Serbia; ajanicijevic@politehnika.edu.rs (A.J.); dkovacevic@politehnika.edu.rs (D.K.); 2 University of Belgrade, Faculty of Mechanical Engineering, Kraljice Marije 16, 11000 Belgrade, Serbia; verapetarpavlovic@gmail.com; 3 Vinca Institute of Nuclear Sciences, National Institute of the Republic of Serbia, University of Belgrade, Mike Petrovica Alasa 12-14, 11000 Belgrade, Serbia; markoperic1983@gmail.com; 4 Department of Mathematics and Physics, North Carolina Central University, Durham, NC 27707, USA; vlahovic@nccu.edu; 5 NASA University Research Center for Aerospace Device Research and Education and NSF Center of Research Excellence in Science and Technology Computational Center for Fundamental and Applied Science and Education, Durham, NC 27707, USA; 6 University of Belgrade, Faculty of Agriculture, Department for Physics and Mathematics, Nemanjina 6, Zemun, 11000 Belgrade, Serbia; 7 Institute of Technical Sciences of SASA, Knez Mihailova 35/IV, 11000 Belgrade, Serbia; suzana.filipovic@itn.sanu.ac.rs

**Keywords:** nanocomposites, polymer synthesis, nanocellulose, Fe_3_O_4_ functionalization, DFT calculation

## Abstract

The rise of innovation in the electrical industry is driven by the controlled design of new materials. The hybrid materials based on magnetite/nanocellulose are highly interesting due to their various applications in medicine, ecology, catalysis and electronics. In this study, the structure and morphology of nanocellulose/magnetite hybrid nanomaterials were investigated. The effect of nanocellulose loading on the crystal structure of synthesized composites was investigated by XRD and FTIR methods. The presented study reveals that the interaction between the cellulose and magnetic nanoparticles depends on the nanocellulose content. Further, a transition from cellulose II to cellulose I allomorph is observed. SEM and EDS are employed to determine the variation in morphology with changes in component concentrations. By the calculation of magnetic interactions between adjacent Fe^3+^ and Fe^2+^ ions within composites, it is determined that ferromagnetic coupling predominates.

## 1. Introduction

Development of hybrid organic/inorganic composites attracts great attention in the scientific community. Hybrid multifunctional materials have advantages, due to the combination of different properties of organic–inorganic ingredients, where an organic component, such as nanocellulose, contributes with high flexibility, dielectric and piezoelectric properties, while an inorganic component may contribute with magnetic and electric properties [1]. Several studies emphasized the importance of achieving improved material properties through the prevention of the mutual inhibition of different origin fillers and the promotion of compatibility at the interface matrix/added components. The resulting hybrid material has demonstrated diverse properties, which can be tailored by changing components, loadings, morphology, and the arrangement of an individual component, etc. [2,3]. As a result, the materials with a broad range of applications, such as drug delivery, magnetic resonance imaging (MRI), sensors, magnetic storage media, photocatalysts and electromagnetic absorption materials, etc., can be developed [4,5,6,7,8,9].

Biosensors are devices that convert (bio)chemical information into an electronic signal by an appropriate transducer containing analyte detection structures. Such a measured signal can be read by an instrument. The major problems for modern biosensors are low reproducibility and the lower stability of the bioreceptor. Thus, cellulose-based materials possess a fine fibrous matrix that is convenient for immobilizing the receptor or nanoparticles, metal oxides and enzymes [10,11].

Nanocellulose (NC) is one of the most frequently used biomasses in nature, because NC is easily degradable, renewable and non-toxic; possesses a high reinforcing strength and stiffness; and is a low-cost material [12]. It should be noticed that NC and its composites are far more often used as a barrier for liquid and gaseous materials, biomedicine, catalysis and water purification [13,14,15] than for electronic or multiferroic applications, despite their good dielectric and piezoelectric properties [16,17].

The main disadvantages of NC for these types of applications are related to its polar and hydrophilic nature, owing to the existence of -OH groups on their surface, which can be avoided by surface modification [18,19]. As a result, controlled modification of the nanocellulose surface offers the possibility for the production of electronic hybrid inorganic−organic nanomaterials. For this type of investigation, the nanocellulose has been coated with various magnetic particles [20,21,22,23,24]. It has been found that the incorporation of magnetite into NC enables selectively targeting, the detection, and potentially the treatment of the cancer tissue via magnetic resonance imaging and inductive heating. This approach can be applied in the biomedical area, for sensors, etc., due to the ferromagnetism and superparamagnetism of small-sized, ferro- and ferrimagnetic iron oxide nanoparticles in the magnetic NC hybrid nanomaterials [20]. It was also demonstrated that the saturation magnetization values of the magnetic NC composites can be connected to the changes in the surface of the NC [18]. Moreover, superparamagnetic-like behavior was observed when iron-oxide nanoparticles were embedded in sodium carboxymethyl cellulose (Na-CMC) [25], or within the core-shell structure of the *β*-cyclodextrin-modified cellulose crystals, CNC@Fe_3_O_4_@SiO_2_ [26]. The use of the core-shell nanomaterials, the Fe_3_O_4_-based layered nanostructures in a biosensor application, is discussed in the literature [27]. It was demonstrated that nanomaterials demonstrate advantages in biosensor efficiency due to a high surface/volume ratio, rapid reaction and lower threshold. MNP-Au was used for the sandwich immunoassay, which could be easily handled by the magnetic field. This approach demonstrates a huge potential for the application of MNP-based composites in biosensors. Moreover, oxidized microcrystalline cellulose may form a complex with Fe^2+^ ions, which can be used as the reinforcement inside the composite materials. As a result, these materials are utilized in the area of magnetographic printing and security paper production [28]. It is important to notice that there are a number of parameters that influence magnetic response and its potential impact on electrical and/or multiferroic properties of the NC/Fe_3_O_4_ hybrid materials. For numerous applications, it is especially important to determine and correlate the structural and morphological properties with bond parameters, as well as magnetic interactions, by calculation exchange coupling constants in hybrid materials. Unfortunately, there are not many reports in the literature that use density functional theory DFT in order to describe the interactions in cellulose-based composites [29]. Moreover, to the best of the authors’ knowledge, a DFT calculation has not yet been performed on the NC/Fe_3_O_4_ composite. These results could be of great importance for the sake of the further development of multicomponent hybrid materials based on NC/Fe_3_O_4_.

Taking all this into account, the aim of this report is to analyze the influence of the different amounts of NC on the crystal structure and morphology of nanosized Fe_3_O_4_ functionalized NC composites. In order to enable further optimization of the magnetic properties of these materials, the theoretical calculation of magnetic interactions and magnetic couplings between iron atoms, within the investigated composites, was performed.

## 2. Materials and Methods

### 2.1. Materials

Commercially available cellulose was used for nanocellulose preparation. Components used for co-precipitation, FeSO_4_ × 7H_2_O and FeCl_3_ × 6H_2_O, were purchased from Merck (Darmstadt, Germany). Deionized water (DW, Sigma Aldrich, Saint Louis, MO, USA), with a resistivity of 18 MΩ cm, was used for NC washing, aqueous solution preparation and for dish washing.

### 2.2. Preparation of NC/Fe_3_O_4_

Nanocellulose (NC) was prepared by the acid hydrolysis (H_2_SO_4_) of commercially available cellulose, according to the established procedure [30]. Obtained NC was separated from the liquid phase by centrifugation and washed with deionized water until pH reached the value of 5.5. The functionalized nanocellulose was prepared by the co-precipitation of Fe(II) and Fe(III) ions in an aqueous solution containing NC with ammonia. NC, 1.5 g, was added to 200 mL of distilled water and stirred for 10 min. FeCl_3_ × 6H_2_O and SO_4_ × 7H_2_O were added to a mixture as sources of ferro and ferri ions, maintaining the ratio 2:1 and heated to 60 °C. Chemical precipitation was achieved by adding 8.0 M of the ammonia solution dropwise with vigorous stirring and at a constant pH of 10. Precipitation was followed with changes of orange color suspension to a black precipitate. Different concentrations of NC were achieved by reducing or increasing the amount of NC in aqueous solutions, while maintaining the same concentrations of Fe(II) and Fe(III) ions. After incubation for 4 h at 60 °C, the mixture was cooled to room temperature with stirring. The resulting NC/Fe_3_O_4_ particles were washed several times with distilled water and ethanol, and afterwards, were dried. The weight ratio of NC in Fe_3_O_4_/NC was 1:0.25 (Fe_3_O_4_/NC1), 1:0.5 (Fe_3_O_4_/NC2), 1:1 (Fe_3_O_4_/NC4) and 1:2 (Fe_3_O_4_/NC8).

### 2.3. Characterization Techniques

X-ray diffraction (XRD) data were obtained using a BRUKER D8 ADVANCE with the Vario 1 focusing primary monochromator (Cu k_α1_ radiation, λ = 1.54059 Å). XRD patterns were obtained over the Bragg angle (2*θ*) range of 10–50°.

Fourier-transform infrared (FTIR) spectra of NC/Fe_3_O_4_ samples were recorded in the transmission mode, within the region 400 to 4000 cm^−1^, using a BOMEM (Hartmann and Braun, Quebec City, QC, Canada) spectrometer with a resolution of 4 cm^−1^. 

The dispersion state of Fe_3_O_4_ on the nanocellulose surface and morphology of final composite films has been investigated by scanning electron microscopy (SEM, Tokyo, Japan, model: JEOL JSM 6610LV).

For the theoretical calculation of magnetic interactions and magnetic couplings between iron atoms in the magnetite structure within investigated composite materials, we performed the Density Functional Theory—Broken Symmetry [31,32,33,34] using B3LYP [35,36] and OPBE [37] functionals and a TZP basis set.

## 3. Results and Discussion

**Figure 1:**
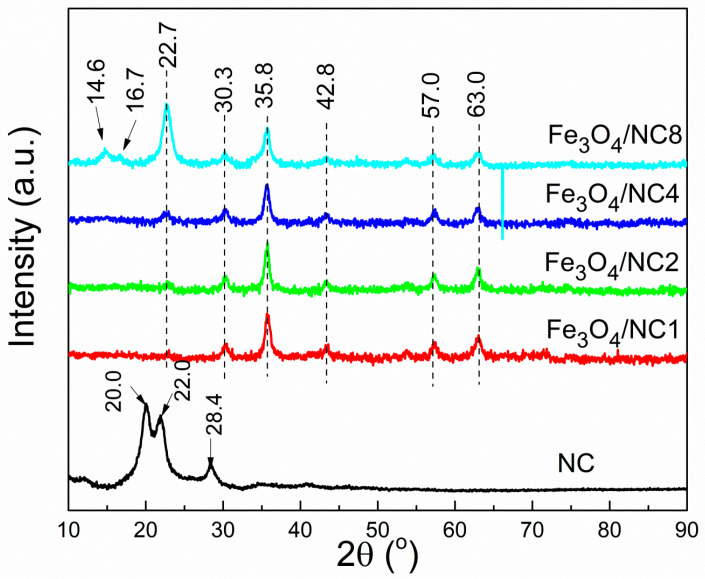
Diffractograms of NC and NC/Fe_3_O_4_ samples.

Changes in the crystal structure and phase purity, due to the variation of NC loadings, are examined by the diffraction of X-rays. The XRD diffractogram of the pure NC and the investigated Fe_3_O_4_/NC composite samples are shown in Figure 1. The diffractogram of the pure NC shows a typical cellulose II structure, with a low intensity peak at 12° and strong doublet of 20 and 22° [38,39]. A similar behavior was obtained by Lani et al. in [40], after the acid hydrolysis of cellulose was isolated from a fruit bunch. With the precipitation of Fe_3_O_4_ in alkali, the conversion of cellulose II into cellulose I occurs. The characteristic signals at 30.3°, 35.8°, 42.8°, 57° and 63° suggest the presence of pure Fe_3_O_4_ with a spinel structure [41] in the Fe_3_O_4_/NC composites. The peak intensities of magnetite slightly decrease with an increase in NC content, due to the decrease of its concentration. The signals corresponding to cellulose are barely noticeable at Fe_3_O_4_/NC ratios of 1:0.25 and 1:0.5, and slightly increase when the content of NC become equal to the content of Fe_3_O_4_. Finally, at the Fe_3_O_4_/NC 1:2 ratio, it becomes the most dominant peak in the graph. It was observed that the signal corresponding to the cellulose is slightly shifted from 22.0 to 22.7° in diffractograms of Fe_3_O_4_/NC, probably due to the coupling of NC with magnetite nanoparticles.

**Figure 2:**
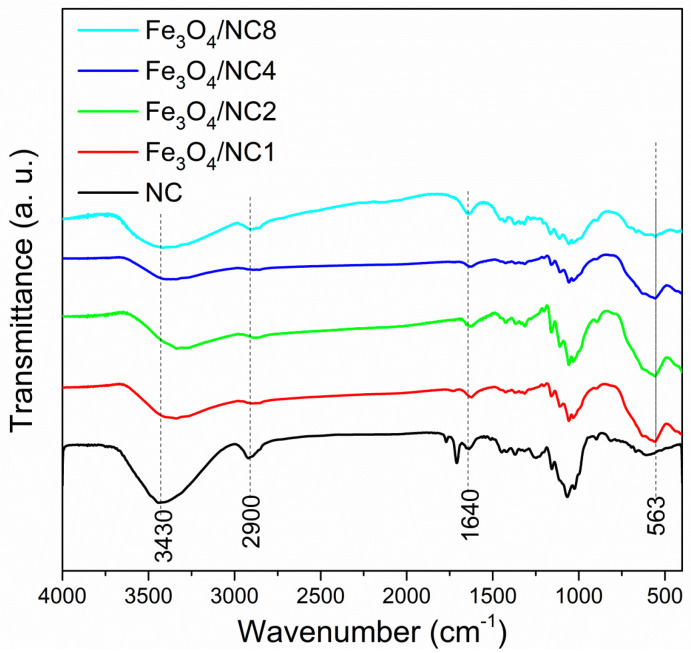
FTIR spectra of of Fe_3_O_4_/NC composites.

Fourier-transform infrared (FT–IR) spectroscopy in the transmission mode was employed to investigate the variation in the infrared optical properties of NC with the precipitation of Fe_3_O_4_ on the nanocellulose surface. The FT-IR transmittance spectra of the NC and composite samples are shown in the Figure 2. The FTIR spectrum of pure nanocellulose was explained in detail in our previous publication [42]. In the spectra of magnetite/NC composites (Figure 2), the bands originating from nanocellulose are the most dominant bands. Characteristic bands, assigned to the O–H stretching vibration at 3430 cm^−1^, C–H stretching vibrations of the –CH_2_ group at 2900 cm^−1^ and –OH bending vibration at 1640 cm^−1^, are observed in the spectra of all samples. A high intensity broad band, at around 563 cm^−1^, is a characteristic band of Fe–O in the tetrahedral sites [43]. Normally, this band appears at around 600 cm^−1^ or a little below this value, as suggested by the literature [44]. A shift of this band to a lower wave number is due to the occurrence of Fe–O bonds on the surface of the nanocellulose. The decrease in the intensity of this signal is caused by the lowering of the Fe_3_O_4_ concentration, from the sample Fe_3_O_4_/NC1 to Fe_3_O_4_/NC8, respectively. The shift of the O–H peak after the deposition of Fe_3_O_4_ on the NC surface suggests the existence of an interaction between the cellulose and magnetic nanoparticles [45].

**Figure 3:**
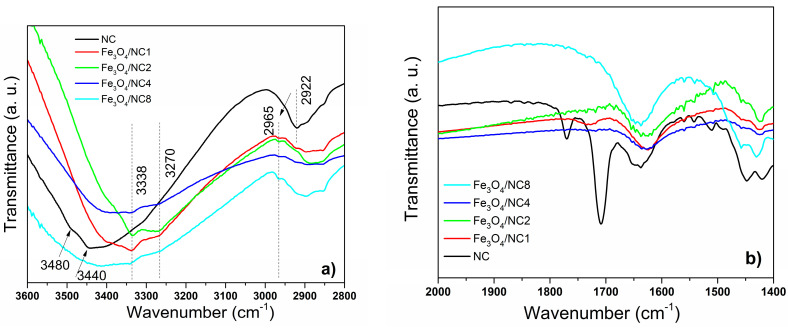
FTIR spectra of the NC and all modified samples: (**a**) 3600–2800 cm^−1^ wavenumber range and (**b**) 2000–1400 cm^−1^ wavenumber range.

The region between 3600 and 2800 cm^−1^ was additionally investigated and depicted in Figure 3a. The maximum absorbance of the OH stretching vibration is positioned at 3440 cm^−1^, indicating intramolecular hydrogen bonding of O2-H---O6 for CN sample. In the cases of the modified samples, the O3-H---O5 and O6-H---O3 intramolecular hydrogen bonding was detected at 3338 cm^−1^ and 3270 cm^−1^, respectively [46]. It was observed that the sample marked as NC exhibits a typical cellulose II structure with a low intensity band at 3480 cm^−1^ and a strong band at 3440 cm^−1^. These bands are characteristic of the cellulose II allomorph, and as it can be noticed from Figure 3a, these bands were not detected in FTIR spectra of the samples modified with Fe_3_O_4_ nanoparticles. In the spectra of the hybrid materials (Fe_3_O_4_/NC1, Fe_3_O_4_/NC2, Fe_3_O_4_/NC4 and Fe_3_O_4_/NC8), bands at 3338 and 3270 cm^−1^ were observed, indicating a transition from cellulose II to cellulose I allomorph, which is in agreement with the results obtained by XRD analysis. Further, the band at 1922 cm^−1^ was significantly shifted to a higher wavenumber after the addition of magnetic particles into the structure, suggesting an exchange in the arrangements caused by the changes of angles around β-1,4-D-glycosidic linkages rearrangement [47].

In the spectra shown in Figure 3b, there are few obvious differences between the NC and modified NC. First of all, the bands at 1720 cm^−1^ and 1510 cm^−1^, which are present in the spectrum of pure nanocellulose, disappear in the spectra of modified hybrid material. According to the literature, these bands originate from the C=C stretching vibration of the aromatic ring and the C=O stretching vibration of carboxylic groups of the lignin and hemicellulose [48]. It was demonstrated earlier that these bands can despair after the treatment of cellulose with NaOH [49]. We have also used an alkali environment during the modification of nanocellulose with magnetic particles, and we consider that as a reason for the disappearance of the earlier mentioned bands. 

The shift to lower wavenumbers and broadening of the FTIR bands usually appears due to a presence of hydrogen bonds between cellulose and filler particles [50]. It was noticed that after the introduction of Fe_3_O_4_ particles, bands of hydroxyl and carboxyl groups were downshifted, indicating a formation of the hydrogen bonds among NC and the Fe_3_O_4_. A shift of the bands at 1640 cm^−1^ and 1420 cm^−1^ was detected in the spectra presented in Figure 3b. With an increasing amount of NC, the shift becomes smaller, and for the sample Fe_3_O_4_/NC8, this shift is almost indistinguishable. This indicates that a lower number of hydrogen bonds between NC and the Fe_3_O_4_ was formed with a decreasing percentage of magnetic nanoparticles in composites. 

**Figure 4:**
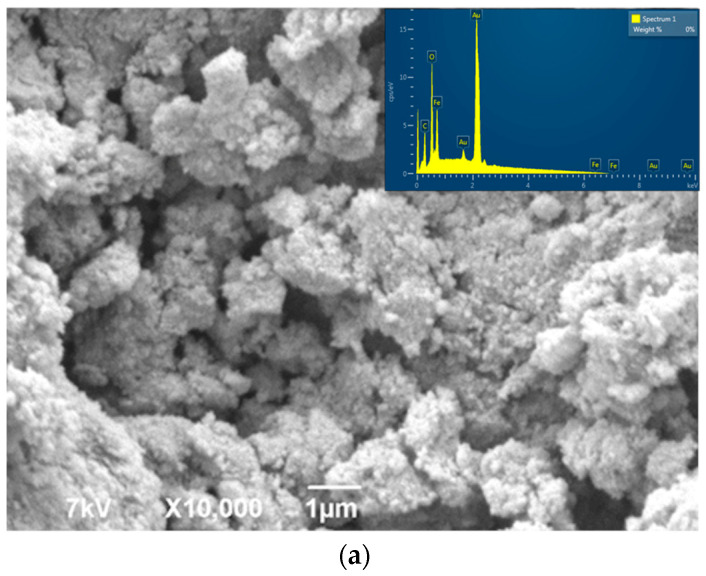

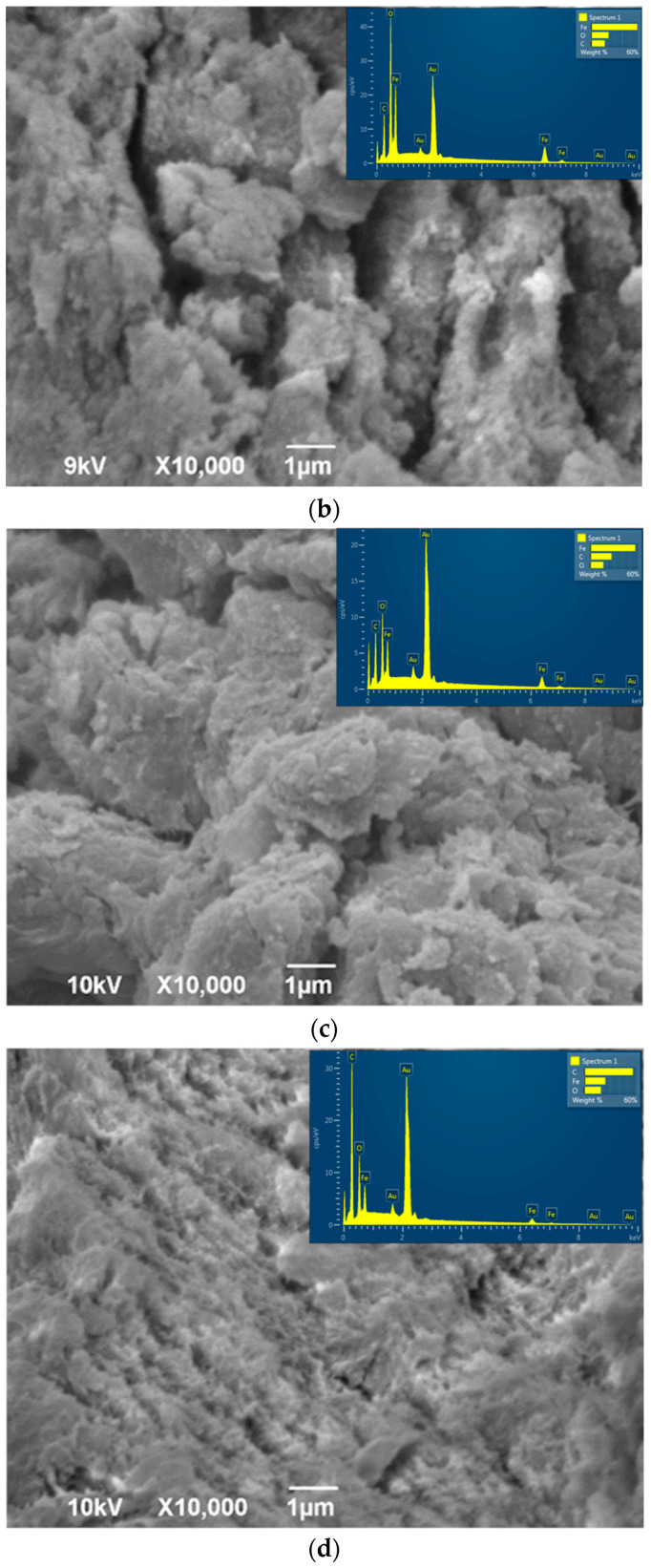
SEM micrographs and EDS of the samples Fe_3_O_4_/NC with different content of NC: (**a**) Fe_3_O_4_/NC1, (**b**) Fe_3_O_4_/NC2, (**c**) Fe_3_O_4_/NC4 and (**d**) Fe_3_O_4_/NC8.

In addition, the SEM analysis was used for a morphological investigation of Fe_3_O_4_/NC (Figure 4). The SEM micrographs indicate that the lower amount of NC resulted in an aggregation and formation of agglomerates of magnetite particles, which are different in size and shape (Figure 4a–c). The irregular Fe_3_O_4_ crystallites grew larger with lower loadings of NC in the composite sample. Apparently, a higher amount of NC favors a more uniform deposition of Fe_3_O_4_ nanoparticles. The homogeneous deposition of Fe_3_O_4_ on the surface of nanocellulose was found for the sample with the highest content of NC (Figure 4d). Generally, it seems that a nanocellulose high-surface area plays a crucial role in their merging into homogeneous layer-like structures.

An energy dispersive X-ray analysis (EDS) was performed at the all Fe_3_O_4_/NC composite samples. The results confirmed the presence of Fe, C and O in the elemental compositions.

An improvement in the homogeneity of the prepared sample continues with an increase in the NC content (Fe_3_O_4_/NC4), i.e., the availability of its larger surface area. Within the Fe_3_O_4_/NC8 sample, the higher substrate area leads to an equal distribution of magnetite nanoparticles on the NC surface. 

Cellulose fibers modified by paramagnetic elements (deposition of iron) may enable an improvement in cellulose orientation in a polymer matrix when exposed to lower strength electric and magnetic fields [28]. Having in mind the significant influence of the NC functionalization with Fe_3_O_4_ and its influence on the magnetic properties of such materials, magnetic interactions in the investigated magnetite structures were analyzed by the determination of exchange coupling constants between neighboring iron atoms. Exchange couplings are described with the model of spin Hamiltonian, namely, the Heisenberg–Dirac–van Vleck (HDVV) Hamiltonian [51,52], which can be expressed by Equation (1):

(1) $${\hat{H}}_{EX}=-J{\hat{S}}_{A}{\hat{S}}_{B}$$

where ${\hat{S}}_{A}$ and ${\hat{S}}_{B}$ stand for the effective spin operators, and *J* represents the exchange coupling constant. For the ferromagnetic interactions, with the parallel spin orientation, *J* has a positive sign, while the negative sign indicates the antiferromagnetic interactions with the antiparallel spin alignment.

The effective spin operators are related to the total spin operator (Equation (2)):

(2) $$\hat{\vec{S}}={\hat{\vec{S}}}_{A}+{\hat{\vec{S}}}_{B}$$

Using the previous two equations, one can rewrite the Heisenberg–Dirac–Van Vleck Hamiltonian in the final form (Equation (3)):

(3) $${\hat{H}}_{EX}=-\frac{J}{2}\left({\hat{\vec{S}}}^{2}-{\hat{\vec{S}}}_{A}^{2}-{\hat{\vec{S}}}_{B}^{2}\right)$$

Hence, the expression for the eigenvalues of *H_EX_* for the different spin states is presented in Equation (4).

(4) $$E\left(\Psi^{2S+1}\right)=-\frac{J}{2}\left[S\left(S+1\right)-S_{A}\left(S_{A}+1\right)-S_{B}\left(S_{B}+1\right)\right]$$

where *S, S_A_* and *S_B_* are the quantum numbers associated with the spin operators, while *E^2S+1^* represents the energy of a spin multiplet.

In order to determine the magnetic couplings between iron atoms in an investigated magnetite structure, we performed the Density Functional Theory—Broken Symmetry (DFT-BS) method developed by Noodleman [31,32,33,34]. The exchange coupling constants were estimated according to the Yamaguchi approach (Equation (5)) [53,54].

(5) $$J=\frac{\left(E_{HS}-E_{BS}\right)}{\langle S^{2}\rangle {}_{HS}-\langle S^{2}\rangle {}_{BS}}$$

where *E_HS_* is the energy of the high spin, *E_BS_* is the energy of the broken symmetry, while $\langle S^{2}\rangle {}_{HS}$ and $\langle S^{2}\rangle {}_{BS}$ are the expectation values of the high-spin and broken symmetry spin operators.

All calculations were conducted using the B3LYP [32,33] hybrid functional and TZP basis set. For comparison purposes, coupling constants were also calculated with the OPBE [37] functional. All calculations were performed with the ORCA program package, version 2.8-20 [55].

**Figure 5:**
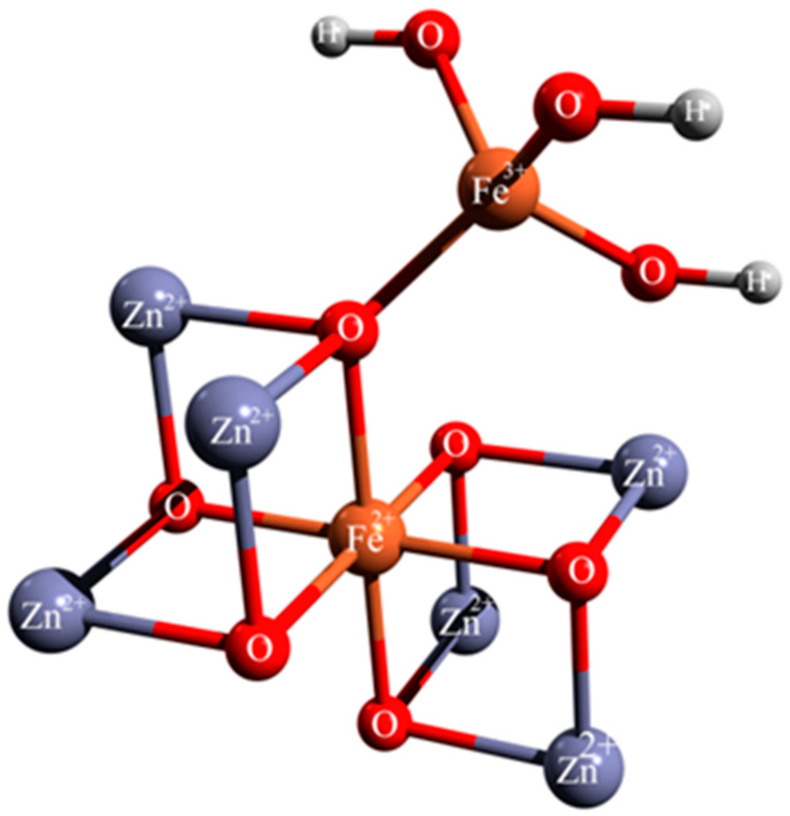
Structure of Fe_3_O_4_ model systems.

The exchange coupling constants between Fe^2+^ and Fe^3+^ ions were calculated on Fe_3_O_4_ model systems, in which the first coordination sphere around the iron atoms was taken directly from the crystal structures (Figure 5). The other atoms were replaced with zinc ions and hydrogen atoms. This represents a very useful method for the calculation of exchange coupling constants in polynuclear metal complexes.

The calculated exchange coupling constants, as well as relevant bond distances, are presented in Table 1.

In all cases, the predominant ferromagnetic coupling was obtained. Although the B3LYP functional represents the method of choice for the calculation of exchange coupling constants, the results obtained with the OPBE functional are quite similar. The calculated values are slightly lower at the B3LYP level of theory, in comparison to the OPBE level of theory. 

**Figure 6:**
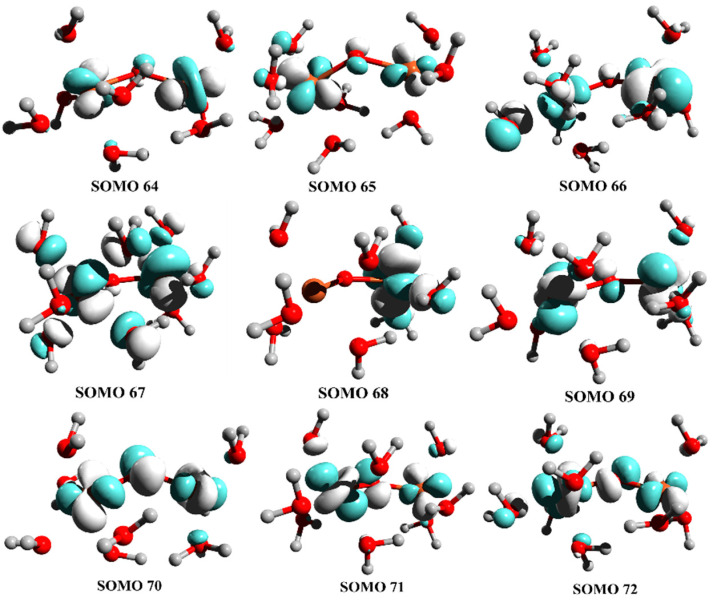
Singly occupied molecular orbitals (SOMO) in high-spin state with S = 9/2. A more simplified structure was used, in which the negative oxygen charge was neutralized by hydrogen atoms.

**Figure 7:**
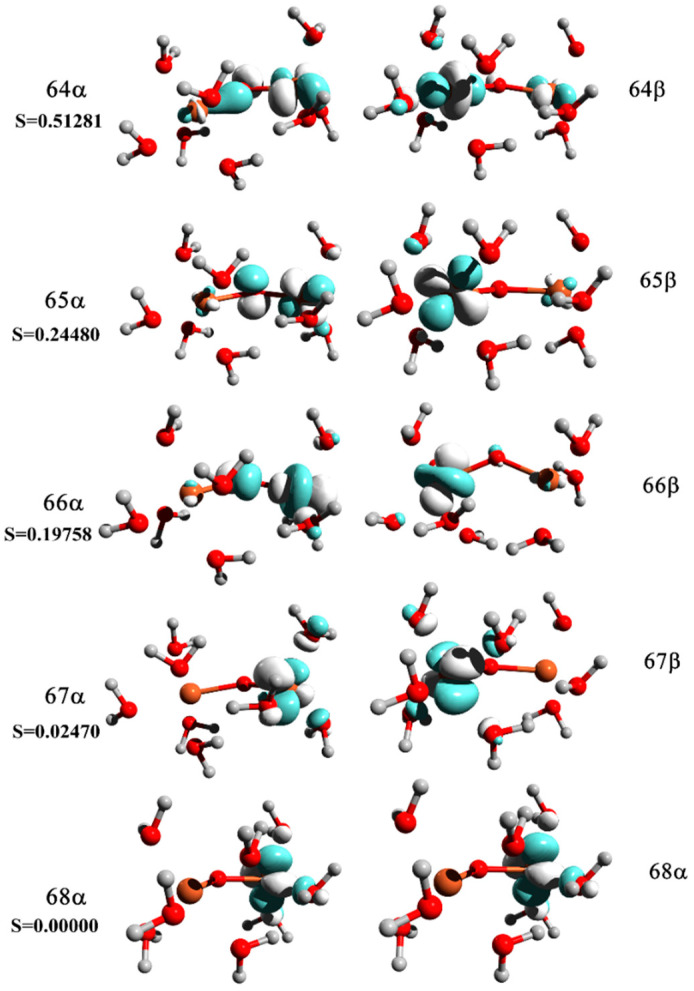
Corresponding magnetic orbitals and spatial overlap integrals, S.

Magnetic couplings between paramagnetic centers are the consequence of the electronic structure and coordination environment around metal ions. In the inverse spinel structure of Fe_3_O_4_, half of the Fe^3+^ ions occupy tetrahedral voids with a high-spin e(2)t_2_(3) electronic configuration, while the Fe^2+^ ions have octahedral coordination with a high-spin t_2g_(4)e_g_(2) electronic configuration. The net interactions between Fe^3+^ ions with tetrahedral coordination and Fe^2+^ ions with octahedral environment can be explained through the analysis of molecular orbitals with predominant metal character (Figure 6 and Figure 7).

Although ferromagnetic couplings have been observed, an orbital analysis indicates the possible antiferromagnetic interactions via a superexchange mechanism, through bridging the oxygen ligand. Moreover, the electron density is delocalized over both metal centers. It is well known that magnetic interactions between paramagnetic centers strongly depend on geometrical parameters, namely on bond distances and angles. In most cases, magnetic interactions decrease with increasing bond distances and angles. The change in the percentage of NC does not drastically influence a change of Fe^2+^-O and Fe^3+^-O bond distances (Table 1). Hence, the differences in magnetic couplings between Fe^2+^ and Fe^3+^ ions in the investigated structures are very small and demonstrate weak dependence on the percentage of NC. In the case of the Fe_3_O_4_/NC1 sample, the Fe^2+^-O and Fe^3+^-O bond distances are slightly longer in comparison to other structures, i.e., 2.081 and 1.846 Å, respectively. Indeed, the ferromagnetic interactions in this case are slightly lower. 

## 4. Conclusions

In this study, the investigation of the influence of a nanocellulose amount on features of hybrid composite materials, based on functionalized nanocellulose with magnetic particles, has been presented. We focused on the investigation of the structural and morphological properties of nanocellulose/magnetite (Fe_3_O_4_/NC) composites. The XRD analysis confirmed the transformation from cellulose II into cellulose I allomorph with the addition of a magnetic particle. Further, a small but noticeable shift of the diffraction line at the Bragg angle of 22° indicates coupling between phases. Detailed FTIR analysis confirmed the results obtained by XRD, i.e., the transition among allomorphs, along with changes in structure due to the covering of NC with Fe_3_O_4_. In order to enable the further development of multicomponent hybrid materials based on NC/Fe_3_O_4_, a theoretical calculation of magnetic interactions and magnetic couplings between iron atoms in the magnetite structure within the investigated composite materials were performed. A B3LYP hybrid functional and TZP basis set were used for the calculation of bond distances and for the determination of coupling constants between neighboring iron atoms in Fe_3_O_4_/NC composites. The derived model confirmed the predominant ferromagnetic coupling. It was demonstrated that the amount of NC has an influence on the bound distance and angles. This conclusion suggests that the NC content may affect magnetic couplings between neighboring Fe^3+^ and Fe^2+^ ions.

This research provides expellant inputs for further investigation in the area of biosensors. The Fe_3_O_4_ component of the composite can be easily manipulated with the permanent magnet, and the NC component is biodegradable and possesses piezoelectric properties. The MNP/NC is comprised of a multifferoic material, which electric and magnetic properties can be tailored to by different amounts of NC, and in that way, this composite is promoted as a good candidate for various sensor applications. We believe this material should be further examined in the domain of its magnetic and electrical properties, as well as for its use as a biosensor for appropriate antigen and antibody systems.

## Figures and Tables

**Table 1:** Calculated exchange coupling constants (cm^−1^) at OPBE and B3LYP level of theory and relevant bond distances (Å) from X-ray data.

Structure	J_OPBE_	J_B3LYP_	Fe^2+^-O	Fe^3+^-O
Fe_3_O_4_/NC1	48.7	46.7	2.081	1.846
Fe_3_O_4_/NC2	49.8	48.1	2.069	1.835
Fe_3_O_4_/NC4	49.3	47.7	2.073	1.839
Fe_3_O_4_/NC8	49.5	47.8	2.071	1.837

## Data Availability

The raw/processed data required to reproduce these findings cannot be shared at this time as the data also form part of an ongoing study.
